# The ins and outs of CO_2_

**DOI:** 10.1093/jxb/erv451

**Published:** 2015-10-14

**Authors:** John A. Raven, John Beardall

**Affiliations:** ^1^Division of Plant Sciences, University of Dundee at the James Hutton Institute, Invergowrie, Dundee DD2 5DA, UK†, and School of Plant Biology, University of Western Australia, M084, 35 Stirling Highway, Crawley, WA 6009, Australia; ^2^School of Biological Sciences, Monash University, VIC 3800, Australia

**Keywords:** Aquaporins, bicarbonate, carbon concentrating mechanisms, C_4_, carbon dioxide, crassulacean acid metabolism, leakage, lipid bilayer, permeability.

## Abstract

It is difficult to distinguish influx and efflux of inorganic C in photosynthesizing tissues; this article examines what is known and where there are gaps in knowledge. Irreversible decarboxylases produce CO_2_, and CO_2_ is the substrate/product of enzymes that act as carboxylases and decarboxylases. Some irreversible carboxylases use CO_2_; others use HCO_3_^–^. The relative role of permeation through the lipid bilayer versus movement through CO_2_-selective membrane proteins in the downhill, non-energized, movement of CO_2_ is not clear. Passive permeation explains most CO_2_ entry, including terrestrial and aquatic organisms with C_3_ physiology and biochemistry, terrestrial C_4_ plants and all crassulacean acid metabolism (CAM) plants, as well as being part of some mechanisms of HCO_3_^–^ use in CO_2_ concentrating mechanism (CCM) function, although further work is needed to test the mechanism in some cases. However, there is some evidence of active CO_2_ influx at the plasmalemma of algae. HCO_3_^–^ active influx at the plasmalemma underlies all cyanobacterial and some algal CCMs. HCO_3_^–^ can also enter some algal chloroplasts, probably as part of a CCM. The high intracellular CO_2_ and HCO_3_^–^ pools consequent upon CCMs result in leakage involving CO_2_, and occasionally HCO_3_^–^. Leakage from cyanobacterial and microalgal CCMs involves up to half, but sometimes more, of the gross inorganic C entering in the CCM; leakage from terrestrial C_4_ plants is lower in most environments. Little is known of leakage from other organisms with CCMs, though given the leakage better-examined organisms, leakage occurs and increases the energetic cost of net carbon assimilation.

## Highlight

CO_2_ supply to Rubisco can involve diffusive CO_2_ fluxes or a CO_2_ concentrating mechanism. The mechanisms involve CO_2_ loss in photorespiration or by leakage, respectively.

## Introduction

The textbook equations for oxygenic photosynthesis and for dark respiration have CO_2_ as, respectively, the inorganic C substrate and the inorganic C product. This is the case for the core autotrophic carboxylase of oxygenic photosynthetic organisms, ribulose-1,5-bisphosphate carboxylase-oxygenase (Rubisco), with CO_2_ as its inorganic carbon substrate, and for the decarboxylases of dark respiration with CO_2_ as their inorganic C product (Raven, 1972a; Table 5.2 of Raven, 1984; Table 3 of Raven, 1997a). Add to this the Overton prediction over a century ago that CO_2_ has a high permeability in lipid bilayers (see Endeward *et al.*, 2014) and it appears at first sight that the textbook equations describe not just the inorganic C substrate for photosynthesis and inorganic C product of dark respiration, but also the inorganic C species crossing cell membranes between the external environments and intracellular sites of inorganic C consumption and production.

However, it is clear that this picture is significantly over-simplified in (at least) two ways. One is that we now know of CO_2_-permeable channels (a subset of the aquaporins, and analogues) in some cell membranes, and there is considerable debate as to their functional significance if the CO_2_ permeability of the lipid bilayer is very high (Boron *et al.*, 2011; Itel *et al.*, 2012; Endeward *et al.*, 2014; Kai and Kaldendorf, 2014). The second over-simplification is that there are known CO_2_ concentrating mechanisms (CCMs), involving active transport of some inorganic C species (or H^+^) and/or C_4_ or crassulacean acid metabolism (CAM) biochemistry, accounting for about half of global primary productivity. This assertion is based on the global net primary productivity values of 56 Pg C per year on land and 49 Pg C per year in the ocean (Field *et al.*, 1998), the assumption that the ratio of global C_4_ gross primary productivity (almost all terrestrial) to total gross primary productivity, i.e. 0.23 (Still and Berry, 2003), also applies to net primary productivity, and the assumption that not less than 0.8 of the marine global net primary productivity is carried out by organisms with CCMs (Raven *et al.* 2012, 2014; Raven and Beardall, 2014). These assumptions give a total CCM-based global net primary productivity of (0.23×56) + (0.8×49) or 52 Pg C per year out of a total of 105 Pg C per year global net primary productivity.

CCMs necessarily involve an energy input to generate a net flux of inorganic C from the environment with a relatively low CO_2_ concentration to the active site of Rubisco where a higher steady-state CO_2_ concentration is maintained during photosynthesis. This means that the direction of the CO_2_ free energy gradient (inside concentration > outside) is the opposite of that for photosynthesis with C_3_ physiology and biochemistry (inside < outside). Accordingly, in an organism expressing a CCM, the high CO_2_ permeability of the pathway from the environment to Rubisco required for high rates of photosynthesis in organisms with C_3_ physiology and biochemistry would result in a decreased net rate of photosynthesis and increased energy requirement per net CO_2_ assimilated (Raven *et al.*, 2014). The final result is an increased energy cost of CCMs relative to that of diffusive entry with C_3_ physiology and biochemistry.

Important progress in understanding bidirectional fluxes of inorganic carbon in an organism expressing a CCM has been made in a recent paper in the *Journal of Experimental Botany* (Eichner *et al.*, 2015). Using the marine diazotrophic cyanobacterium *Trichodesmium*, this work combined two experimental approaches, membrane inlet mass spectrometry to distinguish CO_2_ from HCO_3_^–^ fluxes (Badger *et al.*, 1994) and measurements of the natural abundance of ^13^C relative to ^12^C (Sharkey and Berry, 1985), with modelling. A very important conclusion is that internal cycling of inorganic C is significant for the natural isotope abundance of ^13^C:^12^C in the organism, and for cellular energy budgets. This commentary considers wider aspects of CCMs and of leakage of inorganic carbon from them, and how the findings of Eichner *et al.* (2015) might help further interpretation of data on other organisms, including eukaryotic algae and vascular plants.

## Species of inorganic C involved in carboxylases and decarboxylases

All of the unidirectional decarboxylases examined (functioning far from thermodynamic equilibrium), i.e. those of the tricarboxylic acid cycle and the oxidative pentose phosphate pathway, produce CO_2_ (Raven, 1972a,b). By analogy with such unidirectional decarboxylases, the product of glycine decarboxylase, the enzyme responsible for CO_2_ production in the photorespiratory carbon oxidation cycle, is also very likely to be CO_2_.

Enzymes that function *in vivo* sufficiently close to thermodynamic equilibrium, and hence can function as carboxylases and decarboxylases, both consume and produce CO_2_ (Table 5.2 of Raven, 1984; Häusler *et al.*, 1987; Jenkins *et al.*, 1987; Table 3 of Raven, 1997a). Significantly for the present article, the decarboxylase function of three of these enzymes (phosphoenolpyruvate carboxykinase; NAD^+^ malic enzyme; NADP^+^ malic enzyme) is involved in the decarboxylation step of C_4_ and CAM photosynthesis (Jenkins *et al.*, 1987).

Among unidirectional carboxylases, operating far from thermodynamic equilibrium, a number use CO_2_ as the inorganic C substrate (Table 5.2 of Raven, 1984; Häusler *et al.*, 1987; Jenkins *et al.*, 1987; Table 3 of Raven, 1997a; Firestyne *et al.*, 2009). Importantly for the present article, these CO_2_-consuming carboxylases include Rubisco, the core carboxylase of all oxygenic photosynthetic organisms, as well as the 5-aminoimidazole ribonucleotide carboxylase required for purine synthesis.

Finally, some unidirectional carboxylases consume HCO_3_^–^ (Table 5.2 of Raven, 1984; Table 3 of Raven, 1997a). One of these is phosphoenolpyruvate carboxylase, an essential anaplerotic enzyme in almost all oxygenic photosynthetic organisms (Table 4 of Raven, 1997a) as well as the ‘C_3_ + C_1_’ carboxylase of organisms with C_4_ photosynthesis (with the exception of the ulvophycean marine macroalga *Udotea flabellum*: see Raven, 1997a) and with CAM photosynthesis. A possible alternative ‘C_3_ + C_1_’ carboxylase for C_4_ and CAM photosynthesis is pyruvate carboxylase, which also uses HCO_3_^–^ as the inorganic C substrate (Table 5.2 of Raven, 1984; Table 3 of Raven, 1997a). Other carboxylases using HCO_3_^–^ include acetyl CoA carboxylase used in the synthesis of long-chain fatty acids, and carbamoyl phosphate synthase, essential for citrulline, and hence arginine, synthesis (Table 4 of Raven, 1984; Table 3 of Raven, 1997a).

## CO_2_ permeability of lipid bilayers and the role of CO_2_-conducting aquaporins and analogous protein pores

There is still significant uncertainty as to the mechanism of CO_2_ permeation of biological membranes (Boron *et al.*, 2011; Itel *et al.*, 2012; Endeward *et al.*, 2014; Kai and Kaldendorf, 2014). A particular problem is the role of proteinaceous CO_2_ channels if the intrinsic CO_2_ permeability of the lipid bilayer is very high, although there is evidence of increased photosynthesis and growth in terrestrial C_3_ plants expressing CO_2_-transporting aquaporins (Uehlein *et al.*, 2003, 2008; Heckwolf *et al.*, 2011) The most convincing evidence for the role of aquaporins in terrestrial C_3_ plants comes from Hanba *et al.* (2004) and Tsuchihira *et al.* (2010). Table 1 shows the permeability coefficient for CO_2_ of planar lipid bilayers of various compositions, and for the plasmalemma vesicles derived from high and low CO_2_-grown *Chlamydomonas reinhardtii.* In all three cases attempts were made to eliminate the influence of diffusion boundary layers on each side of the membrane on the measured permeability.

**Table 1.** Permeability coefficient for CO_2_ in planar lipid bilayers and plasmalemma vesicles, corrected as far as possible for limitation by aqueous diffusion boundary layers Also shown are the modelled ‘optimum’ or ‘maximum’ (for functioning in the CCM) CO_2_ permeability of the wall of cyanobacterial carboxysomes and/or the estimated CO_2_ permeability of the wall of cyanobacterial carboxysomes.

**Experimental system**	**CO**_**2**_ **permeability** **m s** ^**–1**^	**References**
Planar lipid bilayer composed of 1:1 egg lecithin:cholesterol. 22–24 °C	3.5±0.4.10^–3^ (standard error)	Gutknecht *et al.* (1977)
Plasmalemma vesicles of *C. reinhardtii* grown photolithtrophically in media with high low (350 μmol mol^–1^ total gas) and high (50 mmol mol^–1^ total gas) CO_2_ for growth.? °C	0.76±0.03–1.49±0.2.10^–5^ (± standard error; low CO_2_-grown cells) 1.21±0.01–1.8±0.17.10^–5^ (± standard error; high CO_2_- grown cells)	Sültemeyer and Rinast (1996)
Planar lipid bilayer composed of (i) pure diphytanoyl-phosphatidyl choline (ii) 3:2:1 cholesterol: diphytanoyl-phosphatidyl choline: egg sphingomyelin, and (iii) mixture of lipids mimicking the red cell plasmalemma.? °C	≥3.2±1.6.10^–2^(not clear what ± refers to; ≥3.2 refers to all three membrane compositions)	Missner *et al.* (2008)
Estimate of upper limit on CO_2_ permeability of cyanobacterial carboxysome wall consistent with CCM function.	10^–7^–2.5.10^–6^	Reinhold et al. (1987, 1991)
Estimate of CO_2_ permeability of the carboxysome wall of *Synechococcus* assuming all of the limitation of CO_2_ efflux from carboxysomes is in the carboxysome wall. 30 °C	2.2.10^–7^ (no estimates of error given)	Salon *et al.* (1996a,b), Salon and Canvin (1997)
Estimate of CO_2_ permeability of the carboxysome wall in *Anabaena variabilis* assuming all of the limitation to CO_2_ efflux from carboxysomes is in the carboxysome wall. 30 °C	2.8±0.8.10^–7^ (standard error, *n*=9)	McGinn *et al.* (1997)
Estimate of ‘optimal’ CO_2_ permeability of cyanobacterial carboxysome wall from CCM model.	10^–5^	Mangan and Brenner (2014)
CO_2_ permeability of carboxysome wall in *Prochloroccus* estimated from a model of CCM function.	10^–7^	Hopkinson *et al.* (2014)
Estimate of CO_2_ permeability of the carboxysome wall in *Prochlorococcus* assuming all of the limitation to CO_2_ efflux from carboxysomes is in the carboxysome wall.	10^–6^	Hopkinson *et al.* (2014)

Method for all three data sets involves measurement of inorganic carbon fluxes, expressed as CO_2_, under a known CO_2_ concentration difference across the membrane across planar membrane bilayers (Gutknecht *et al.*, 1997; Missner *et al.*, 2008) or plasmalemma vesicles of *Chlamydomonas* (Sültemeyer and Rinast, 1996). Carbonic anhydrase was added to both sides of the membrane to minimize the gradient of CO_2_ across the aqueous diffusion boundary layers on each side of the membrane.

## CO_2_ entry in organisms lacking a biophysical CCM

The classic example of these is the C_3_ vascular land plants. It is now clear that CO_2_ is the species of inorganic carbon entering the cells from the cell wall (Colman and Espie, 1985; Espie and Colman, 1986; Espie *et al.*, 1986; Evans *et al.*, 2009; Maberly, 2014). The assumption is that terrestrial C_4_ and CAM vascular plants also rely on CO_2_ entry from the cell wall to the cytosol where carbonic anhydrase equilibrates CO_2_ with HCO_3_^–^, the inorganic C substrate for PEPc (Colman and Espie, 1985; Nelson *et al.*, 2005).

For C_3_ plants the transport of CO_2_ from the outside of the cell wall to Rubisco involves diffusion of CO_2_ across the plasmalemma and across the outer and inner chloroplast membranes and, in the aqueous phase, through the cell wall, the cytosol and the stroma (Colman and Espie, 1985). It is implicitly assumed that there is no carbonic anhydrase in the cell wall (Raven and Glidewell, 1981; Colman and Espie, 1985) of C_3_ plants, though there seems to be no experimental evidence demonstrating this. Carbonic anhydrases could equilibrate CO_2_ and HCO_3_^–^ in the cytosol and stroma and so enlist the predominant (at the pH of the cytosol and stroma) inorganic species, HCO_3_^–^, in CO_2_ transport across these aqueous phases, with the required H^+^ flux carried inwards by protonated buffers with a return flux outwards of the deprotonated buffers (Raven and Glidewell, 1981; Colman and Espie, 1985; Evans *et al.*, 2009; Niinemets *et al.*, 2009; Tazoe *et al.*, 2009, 2011). There is very significant interspecific variation in the magnitude of the mesophyll conductance (= permeability) of C_3_ seed plants (Evans *et al.*, 2009; Niinemets *et al.*, 2009; Tazoe *et al.*, 2009; see Table 2).

**Table 2.** Permeability coefficient (‘mesophyll conductance’) for CO_2_ entry for the pathway from the outside of the external aqueous diffusion boundary layer to Rubisco in C_3_ biochemistry No data seem to be available for the corresponding CO_2_ movement to PEPc in C_4_ or CAM biochemistry.

Category of plant: flowering plant, hornwort, or liverwort	Mesophyll permeability m s^–1^
Herbaceous dicotyledonous flowering plant	2.16±0.32.10^–4^ (standard error, *n* not clear)
Herbaceous monocotyledonous flowering plant	2.24±0.29.10^–4^ (standard error, *n* not clear)
Woody deciduous dicotyledonous flowering plant	1.05±0.12.10^–4^ (standard error, *n* not clear)
Woody evergreen dicotyledonous flowering plant	0.85±0.08.10^–4^ (standard error, *n* not clear)
Hornwort	1.75.10^–4^ (no statistics provided by Meyer *et al.*, 2008)
Unventilated liverwort	1.90±0.15.10^–4^ (standard deviation, *n*=3
Ventilated liverwort	0.80±0.04.10^–4^ (standard deviation, *n*=3)

Conversion of photosynthetic rates for the plants on a projected leaf area basis (from Table 1 of Warren, 2008) to the area of mesophyll cells exposed to the intercellular gas space uses a ratio of 25 m^2^ m^2^ mesophyll cells exposed to the intercellular gas space projected leaf area (from pp. 380–381of Nobel, 2005). Conversion of the difference in CO_2_ concentration between the outside of the cell wall to the chloroplast stroma expressed in terms of atmospheric mol fraction (μmol CO_2_ mol^–1^ total atmospheric gas) from Table 1 of Warren (2008) to mmol CO_2_ dissolved in each m^3^ of leaf water uses a conversion factor of 1 mmol CO_2_ m^–3^ dissolved in leaf water for each 20.4 μmol CO_2_ mol^–1^ total atmospheric gas (from pp. 377 and 384 of Nobel, 2005). For a ventilated thalloid liverwort the ratio of 9 m^2^ mesophyll cells exposed to the intercellular gas space per m^2^ projected thallus area (Green and Snelgar, 1982), and for a hornwort or and unventilated liverwort thallus the ratio is 1 (Green and Snelgar, 1982), with other data from Meyer *et al.* (2008).

Turning to submerged aquatic organisms, a number have CO_2_ entry followed by diffusive flux to Rubisco, resembling C_3_ land plants, although aquatic vascular plants lack stomata. These organisms include a number of freshwater and marine algae, aquatic bryophytes, and freshwater vascular plants (Raven, 1970; MacFarlane and Raven, 1985, 1989, 1990; Kübler *et al.*, 1999; Sherlock and Raven, 2001; Maberly and Madsen, 2002; Raven *et al.*, 2005; Maberly *et al.*, 2009; Maberly, 2014). While these organisms share some of the physiological characteristics found in organisms with CCMs, e.g. the absence of a competitive interaction between CO_2_ and O_2_ in photosynthetic gas exchange (Kübler *et al.*, 1999; Sherlock and Raven, 2001; Maberly *et al.*, 2009), the overall influence of environmental factors points to diffusive CO_2_ entry.

## CO_2_ entry in organisms expressing a biophysical CCM

A biophysical CCM that involves diffusive CO_2_ entry was first proposed by Walker *et al.* (1980; see Briggs, 1959) for ecorticate giant internodal cells of freshwater green algal macrophytes of the Characeae growing in relatively alkaline waters. The localized active efflux of H^+^ across the plasmalemma causes a localized decrease in pH in the cell wall and diffusion boundary layer, to approximately 2 pH units below that in the medium. As HCO_3_^–^ diffuses into the acid zone, the equilibrium CO_2_:HCO_3_^–^ increases 100-fold, as does the rate of HCO_3_^–^ conversion to CO_2_ in the absence of carbonic anhydrase (Walker *et al.*, 1980). Subsequently, expression of carbonic anhydrase in the acid zones was demonstrated, further increasing the rate of HCO_3_^–^ to CO_2_ conversion (Price *et al.*, 1985; Price and Badger, 1985). Intracellular acid-base regulation requires alkaline zones between the acid zones. This mechanism also occurs in some freshwater flowering plants where the acid zone is on the abaxial leaf surface and the alkaline zone is on the adaxial leaf surface (Maberly and Madsen, 2002). The high CO_2_ concentration generated in the acid zones can, after crossing the plasmalemma by diffusion, give an internal CO_2_ concentration rather less than that in the acid zone but still sufficient to constitute a CCM with the CO_2_ concentration inside the cell higher than that in the bulk medium (Walker *et al.*, 1980; Price *et al.*, 1985; Price and Badger, 1985). As well as CO_2_ leakage from the acid zones to the bulk medium, CO_2_ could also leak from the cytosol back to the bulk medium through the alkaline zones.

A similar mechanism is thought to occur in many marine macrophytes as a mechanism of using external HCO_3_^–^ (Raven and Hurd, 2012). However, the evidence for this is (a) inhibition of external HCO_3_^–^ use by pH buffers that, ex hypothesis, eliminate the acid zones, (b) inhibition of external carbonic anhydrase using a membrane-impermeant inhibitor as well as, in some cases, (c) showing that photosynthesis is not decreased by inhibitors of one group of plasmalemma HCO_3_^–^ transporters (Raven and Hurd, 2012). There have been no direct demonstrations of the acid zones in macroalgae because, although they can be visualized when they occur in the freshwater Characeae and vascular macrophytes, they must (if they exist!) occupy smaller areas in marine macro algae and in seagrasses (Raven and Hurd, 2012).

An analogous mechanism involves external HCO_3_^–^ entry at the plasmalemma and across the chloroplast envelope membranes, not necessarily giving higher internal than external concentration, with HCO_3_^–^ entry to the thylakoid lumen via (ex hypothesis) HCO_3_^–^-transporting channels (Raven, 1997b; Jungnick *et al.*, 2014; Raven *et al.*, 2014). The low pH of the thylakoid lumen, with the presence (at least in *Chlamydomonas*) of a carbonic anhydrase, gives a rate of CO_2_ production, and an equilibrium CO_2_ concentration, similar to that in the extracellular acid zones of some freshwater macrophytes (Raven, 1997b; Moroney and Ynalvez, 2007; Jungnick *et al.*, 2014; Raven *et al.*, 2014). The final step in the CCM is diffusion of the CO_2_ from the lumen to the stroma, and especially to the pyrenoid where most of the Rubisco occurs in *Chlamydomonas* (Raven, 1997b; Raven *et al.*, 2014). CO_2_ leakage could occur from the pyrenoid back to the bulk medium.

**Fig. 1.**
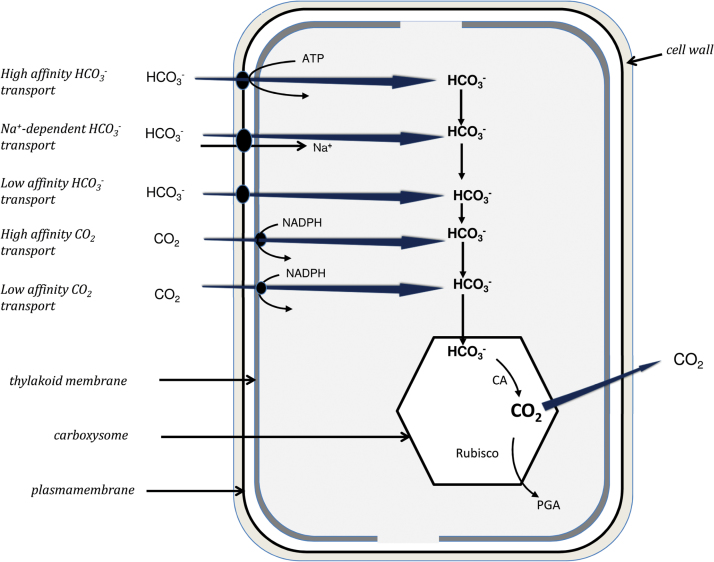
A schematic model for inorganic carbon transport, and CO_2_ accumulation and leakage in cyanobacteria. Low affinity transport systems are shown in grey and high affinity systems are shown in black, and are found at the plasmalemma and/or thylakoid membrane. Transporters whose characteristics are unknown are shown in white. Redrawn after Fig. 1 of Price *et al.* (2002), Badger and Price (2003), and Giordano *et al.* (2005). (Price *et al.* 2002. Modes of active inorganic carbon uptake in the cyanobacterium Synechocystis sp. PCC7942. Functional Plant Biology 29, 131–149. CSIRO PUBLISHING (http://www.publish.csiro.au/nid/102/paper/PP01229.htm). (Badger and Price 2003. CO_2_ concentrating mechanism in cyanobacteria: molecular components, their diversity and evolution. Journal of Experimental Botany 54, 609–622). (Giordano *et al.* 2005. CO_2_ concentrating mechanisms in algae: mechanisms, environmental modulation, and evolution. Annual Review of Plant Biology 6, 99–131).

Energetically downhill entry of CO_2_ as part of a CCM occurs in cyanobacteria, although without localized surface acidification (see data of Maeda *et al.*, 2002, and models of Mangan and Brenner, 2014; Eichner *et al.*, 2015) (Fig. 1). Three further essential components are, first, active HCO_3_^–^ influx at the plasmalemma, and the unidirectional conversion of CO_2_ to HCO_3_^–^ energized by the NDHI_4_ component of cyclic electron flow round photosystem I at the outer surface of the thylakoid membrane. Second, the carboxysomes, containing Rubisco and carbonic anhydrase, whose protein subunit walls probably have a limited permeability to CO_2_ (see estimates in Table 1). The cytosolic HCO_3_^–^ from these two sources enters carboxysomes through pores also allowing permeation of anions (Raven, 2006) and H^+^ (Menon *et al.*, 2010) or, perhaps, OH^–^. Finally, HCO_3_^–^ in the carboxysome lumen is acted on by carbonic anhydrase, producing CO_2_ that is (mainly) consumed by Rubisco in the carboxysome, though some CO_2_ could leak to the cytosol. The extent of CO_2_ leakage through the carboxysomal wall is likely to be significant, even with a low CO_2_ permeability coefficient across the carboxysomal wall with its positively charged pores, because of the large CO_2_ accumulation factor (two to three orders of magnitude) in the carboxysome lumen relative to the cytosol during photosynthesis (Eichner *et al.*, 2015). Modelling by Mangan and Brenner (2014) finds that the optimal carboxysome wall permeability coefficient for CO_2_ for maximal CO_2_ accumulation in the carboxysome lumen is around 10^–5^ m s^–1^.

**Fig. 2.**
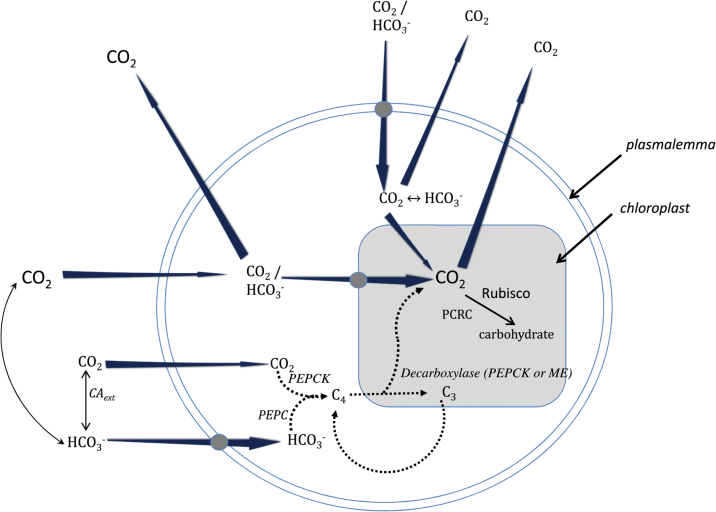
A schematic model for inorganic carbon transport, and CO_2_ accumulation and leakage in eukaryotic algal cells. The model incorporates the possibilities for DIC transport at the plasmalemma and/or chloroplast envelope as well as a putative C_4_-like mechanism. Active transport processes (shown by the shaded boxes) can be of CO_2_ or HCO_3_−. No attempt has been made to show the roles of the various internal CAs in the different compartments. For this the reader is referred to Giordano *et al.* (2005). Redrawn after Giordano *et al.* 2005. CO_2_ concentrating mechanisms in algae: mechanisms, environmental modulation, and evolution. Annual Review of Plant Biology 6, 99–131.

Hopkinson *et al.* (2011) and Hopkinson (2014) propose a general similar mechanism for diatoms, with active HCO_3_^–^ influx at the plasmalemma (Nakajima *et al.*, 2013) and parallel non-energized CO_2_ influx (Fig. 2). These models involve cytosolic carbonic anhydrase to convert the CO_2_ to the equilibrium concentration of HCO_3_^–^, with active HCO_3_^–^ uptake by chloroplasts. This latter step has not yet been identified in diatoms.

Is there a role for active transport of CO_2_? The occurrence of a CO_2_-stimulated ATPase from the ‘microsomal’ fraction (= plasmalemma?) of the freshwater green (chlorophycean) alga *Eremosphaera viridis*, the predominance of CO_2_ uptake in photosynthesis in this alga, and the electroneutrality of CO_2_ uptake (ruling out cation symport), is consistent with CO_2_ uptake by primary active transport (Rotatore *et al.*, 1992; Deveau *et al.*, 1998; Huertas *et al.*, 2000a; Deveau *et al.*, 2001). No other CO_2_ transporters that could reasonably function in active CO_2_ transport are known. Accordingly, the possibility that other eukaryotes depend on a mechanism of the kind suggested by Beardall (1981) and Hopkinson *et al.* (2011), involving passive CO_2_ entry at the plasmalemma and active HCO_3_^–^ transport into the chloroplasts, cannot be ruled out for algae with a CCM and dominant CO_2_ uptake, e.g. acidophilic eukaryotic algae, unless it has been shown that there is no HCO_3_^–^ transporter at the chloroplast envelope. Rotatore and Colman (1990) showed that isolated chloroplasts of *Chlorella ellipsoidea* could take up HCO_3_^–^ by active transport, but had no active CO_2_ uptake; however, the HCO_3_^–^ influx uptake by isolated chloroplasts is less than that at the plasmalemma on a per cell basis (Rotatore and Colman, 1991a,b). Rotatore and Colman (1991c) suggest that there is active uptake of CO_2_ at the plasmalemma of *Chlorella saccharophila* and *C. ellipsoidea*, although the possibility suggested by Beardall (1981) and Hopkinson *et al.* (2011) of passive CO_2_ followed by active entry of inorganic C into chloroplasts cannot be ruled out.

## HCO_3_^–^ entry in organisms expressing a biophysical CCM

Use of HCO_3_^–^ is indicated by more rapid photosynthesis than can be accounted for by the uncatalysed rate of HCO_3_^–^ to CO_2_ conversion (Briggs, 1959). The ‘direct’ use of HCO_3_^–^ involves influx of the anion at the plasmalemma, as compared with the ‘indirect’ use by external conversion to CO_2_ as described in the previous section (see Briggs, 1959). The physiological methods of demonstrating direct use of HCO_3_^–^ involve the known absence, or inhibition, of external carbonic anhydrase(s). In cyanobacteria (Eichner *et al.*, 2015; Hopkinson *et al.*, 2014) and eukaryotic algae such as diatoms (Nakajima *et al.*, 2013), HCO_3_^–^ entry has been shown by physiological methods, and also by molecular genetic techniques, including ectopic expression and tests of functionality of the HCO_3_^–^ transporter gene. The processes in cyanobacteria (Mangan and Brenner, 2014; Eichner *et al.*, 2015) (Fig. 1) and diatoms (Hopkinson *et al.*, 2011; Hopkinson, 2014) (see Fig. 2) have been recently modelled.

In other algae HCO_3_^–^ entry has been shown by physiological methods, including the absence of inhibition of photosynthesis by pH buffers or by inhibition of external carbonic anhydrase, and inhibition by inhibitors of anion exchange proteins (Raven and Hurd, 2012). In some cases, e.g. the eustigmatophycean marine microalga *Nannochloropsis gaditana*, all of the inorganic carbon entering in the CCM involves direct entry of HCO_3_^–^ (e.g. Munoz and Merrett, 1989; Huertas and Lubián, 1997; Huertas *et al.*, 2000b, 2002). In most cases, there is entry of both CO_2_ and HCO_3_^–^ in CCMs (Korb *et al.*, 1997; Tortell *et al.*, 1997; Burkhardt *et al.*, 2001; Giordano *et al.*, 2005; Rost *et al.*, 2006a,b, 2007; Tortell *et al.*, 2008) and, in a few cases (see above) only CO_2_ enters in algae with CCMs.

Isolated, metabolically active chloroplasts of some green algae with CCMs show CO_2_ and HCO_3_^–^ uptake into the chloroplasts as well as into whole cells (Amoroso *et al.*, 1998, van Hunnik *et al.*, 2002, Giordano *et al.*, 2005, and references therein). Yamano *et al.* (2015) show co-operative expression of the plasmalemma HCO_3_^–^ HLA3 ABC transporter and the chloroplast envelope LCIA formate/nitrite transporter homologue (Wang and Spalding, 2014) in *C. reinhardtii.* LCIA is probably a HCO_3_^–^ channel (Wang and Spalding, 2014; Yamano *et al.* 2015); Wang and Spalding (2014) point out that such a channel could not act to accumulate HCO_3_^–^ in the stroma relative to the cytosol since the electrical potential difference across the chloroplast envelope is stroma negative relative to the cytosol.

## Leakage from the intracellular inorganic C pool of CCMs

Significant attention has been paid to leakage of CO_2_ from terrestrial C_4_ plants; this has been thoroughly reviewed by Kromdijk *et al.* 2014 (Table 3 and Supplementary Table S1, available at *JXB* online). For typical C_4_ anatomy with mesophyll cells with intercellular gas spaces and a single bundle sheath layer with limited exposure to intercellular gas spaces and, in some cases, a suberin (mestome) sheath that could further limit CO_2_ leakage, Kromdijk *et al.* (2014) give an excellent critique of the methods used to determine the leakiness to CO_2_ (CO_2_ efflux as a fraction of gross CO_2_ influx) and list their outcomes. These are ^14^CO_2_ labelling to determine the size of the bundle sheath inorganic carbon pool (and hence CO_2_ efflux) or to directly estimate CO_2_ efflux, the deviation of the quantum yield of CO_2_ assimilation from the value predicted from biochemistry assuming no CO_2_ leak, and the natural abundance of stable carbon isotopes in the organic matter of the plant (determined by destructive sampling, or from online measurements of CO_2_ before and after gas flow over photosynthesizing plants) relative to that of source CO_2_. All of these methods have problems (Kromdijk *et al.*, 2014; von Caemmerer *et al.*, 2014). The values for leakiness vary between –0.03–0.70. For the much less common case of terrestrial single-cell C_4_ photosynthesis, the leakiness is similar to that for typical C_4_ anatomy determined by similar methods (King *et al.*, 2012). The permeability of the bundle sheath cells for CO_2_ (1.6–4.5.10^–6^ m s^–1^: Table 4), derived from the CO_2_ efflux from the pool accumulated by the CCM and the driving force of the difference in CO_2_ concentration between the CCM pool and the medium, is at least an order of magnitude higher than the permeabilities for cyanobacteria (Table 4). However, the bundle sheath permeability is two orders of magnitude less than the mesophyll permeability in C_3_ plants (Table 2).

**Table 3.** Leakage of inorganic C from CCMs as a fraction of the inorganic C pumped into the intracellular pool for in terrestrial C_4_ flowering plants, hornworts, eukaryotic algae, and cyanobacteria Values are from Supplementary Table S1 except for C_4_ terrestrial flowering plants where the more detailed data in Table 1 of Kromdijk *et al.* (2014) was used. For C_3_ plants, leakage of CO_2_ from photorespiration is <0.2 of gross CO_2_ fixation (see text).

Organism	Range of CO_2_ leakage estimates as a fraction of gross CO_2_ entry, from Supplementary Table S1	Mean leakage from estimates in Supplementary Table S1 or (C_4_ terrestrial flowering plants) the more detailed data in Table 1 of Kromdijk *et al.* (2014)
C_4_ terrestrial flowering plants	–0.03–0.70	0.260±0.108 (standard deviation, *n*=20)^1^
Hornworts with CCMs	0.170, 0.304, 0.31	0.263±0.066 (standard deviation, *n*=3)^2^
Eukaryotic algae	0.01–0.80	0.36±0.16 (standard deviation, *n*=14): using results from MIMS only.^3^
Cyanobacteria	0.09–0.78	0.407±0.214 (standard deviation, *n*=5): using results from MIMS only.^3^

^1^Calculated from sum of means of ranges in Table 1 of Kromdijk *et al.* (2014), using data from all methods of estimation. Where values are given for more than one irradiance the value from the highest irradiance was used. The theroretically impossible value of –0.03 of leakage obtained by the quantum yield methods was retained rather than being rounded to zero; this made no difference to the outcome.

^2^Estimates from C isotope method, acknowledging that the pyrenoid-based CCM in hornworts may be subject to over-estimation as a result of internal recycling discussed for eukaryotic algae (see Wang and Spalding, 2014).

^3^Estimates from the C isotope method for leakage from a cyanobacterium in excess of 1.0 are theoretically impossible; these and other very high values obtained by this method for the cyanobacteria, are not given here. Possible reasons for these very high values are discussed by Eichner *et al.* (2015). For eukaryotic algae an analogous over-estimate of leakage using the C isotope method to that suggested for cyanobacteria could also occur, at least in *Chlamydomonas* (Wang and Spalding, 2014), but in the case of the eukaryotic algae none of the leakage estimates from using the C isotope method in Supplementary Table S1 are higher than the highest estimates from the MIMS method.

**Table 4.** Permeability coefficients, on a cell surface area basis, for CO_2_ and HCO_3_^–^ determined for efflux of inorganic carbon from the intracellular pool accumulated by CCMs in cyanobacteria and for the bundle sheath of C_4_ plants

Organism	Inorganic carbon species	Permeability coefficient m s^–1^	Reference
*Synechococcus* (Cyanobacterium)	CO_2_	10^–7^ m s^–1^ (no estimates of errors given)	Badger *et al.* (1985)
*Synechococcus*	CO_2_	2.49±0.13.10^–8^ m s^–1^ (standard error, n=4) –3.36±0.14.10^–8^ m s^–1^ (standard error, *n*=18)	Salon *et al.* (1996a,b)
*Synecchococcus* ^1^	HCO_3_^-^	1.47±0.23.10^–9^ m s^–1^ (standard error, n = 7) –1.84±0.17.10^–9^ m s^–1^ (standard error, *n*=7)	Salon *et al.* (1996a,b); Salon and Canvin (1997)
*Anabaena variabilis* (Cyanobacterium)	CO_2_	9.8±1.5.10–^8^ m s^–1^ (standard error, *n*=10)	McGinn *et al.* (1997)
*Anabaena variabilis* ^1^	HCO_3_^−^	7.6±0.9.10^–9^ m s^–1^ (standard error, *n*=7)	McGinn *et al.* (1997)
C_4_ terrestrial flowering plants (5 species)	CO_2_	1.6–4.5.10^–6^ m s^–1^ (no estimates of errors given)	Furbank *et al.* (1989)

^1^The quantification of the efflux of HCO_3_^–^ is less direct than that of CO_2_ efflux. As mentioned by Salon *et al.* (1996b), the permeability coefficient for HCO_3_^–^ is a minimal value since the inside-negative electrical potential difference across the plasmalemma is not accounted for in the calculations.

Leakage of an increased fraction of the CO_2_ released into the bundle sheath by the biochemical CO_2_ pump at low light is thought to be a reason for the rarity of shade-adapted C_4_ plants (see Bellasio and Griffiths, 2014). Bellasio and Griffiths (2014) point out that there is an ontogenetic shading of older leaves in high light-adapted C_4_ plants, and that up to 50% of C_4_ crop photosynthesis occurs in shaded leaves, and investigated CO_2_ leakage in shade-acclimated leaves of the sun-adapted *Zea mays.* They found that CO_2_ leakage as a fraction of PEPc activity (= biochemical CO_2_ pump) stayed constant with decreasing light, thus differing from expectation of a relative increase in leakage. The basis for the this constancy is a decreased PEPc activity relative to that of Rubisco, and fixation of an increased fraction of the CO_2_ generated from respiration in bundle sheath cells.

Less attention has been paid to leakage of CO_2_ from terrestrial CAM plants (Cockburn *et al.*, 1979; Winter and Smith, 1996; Nelson *et al.*, 2005; Nelson and Sage, 2008; Winter *et al.*, 2015).

Cockburn *et al.* (1985) examined the shootless orchid *Chiloschista usneoides* where CAM occurs (in the absence of other photosynthetic structures) in the astomatous velameniferous root. The absence of stomata means that the usual terrestrial CAM method of diurnal closure of stomata decreasing CO_2_ leakage during deacidification and CO_2_ refixation by Rubisco is unavailable. Cockburn *et al.* (1985) showed that the intercellular CO_2_ concentration during deacidification is not significantly different from that of the surrounding atmosphere, while the intercellular CO_2_ concentration during dark acidification is lower than that of the surrounding atmosphere. While lower intercellular CO_2_ concentration in the deacidification phase than is the case of stomata-bearing CAM structures decreases the leakage of CO_2_ from intercellular gas spaces in the stomata-less roots, it also means that carboxylase activity of Rubisco is likely to be substantially below saturation, and the Rubisco oxygenase activity is likely to be significant.

For aquatic vascular plants with CAM there is also no possibility of stomatal limitation of leakage of CO_2_ produced during deacidification in the light. For isoetids there is very little loss from the possible leakage of CO_2_ from the astomatal, cuticularized, photosynthetic part of the leaf, and even loss from any lower, less cuticularized, part of the leaf might be limited or abolished by the high CO_2_ concentration in the surrounding sediment that contains mineralizing particulate organic matter derived by sedimentation from the plankton. The non-isoetid submerged aquatic CAM flowering plant *Crassula helmsii* also lacks the leakage-limiting stomatal closure mechanism of terrestrial *Crassula* spp. *C. helmsii* can show net CAM fixation from external CO_2_ in the dark and also net photosynthetic C_3_ CO_2_ assimilation from external CO_2_ in parallel with refixation of internal CO_2_ generated in deacidification from malic acid, with, presumably, implications for CO_2_ leakage (Newman and Raven, 1995; Maberly and Madsen, 2002; Klavsen and Maberly, 2010).

Of course, the great majority of aquatic primary producers carrying out almost all of the aquatic primary productivity involving CCMs do not express CAM. Essentially all of the work on leakage of CO_2_ from intracellular pools of the CCM in aquatic organisms comes from cyanobacteria and eukaryotic microalgae; very little is known of leakage of CO_2_ from algal macrophytes or submerged aquatic vascular macrophytes that concentrate CO_2_ by C_4_ metabolism or a biophysical CCM. Perhaps the clearest example of CO_2_ leakage comes from the work of Tchernov *et al.* (1997, 1998, 2003) using membrane inlet mass spectrometry (MIMS). This method gives estimates of changes in CO_2_ and O_2_ in solution, with the difference between the two (if the photosynthetic quotient is assumed to be 1) representing the HCO_3_^–^ flux, typically (see above) HCO_3_^–^ influx. Tchernov *et al*. (1997, 1998, 2003) found an increase in external O_2_ and also CO_2_, with the computed HCO_3_^–^ influx exceeding the organic carbon production rate computed from O_2_ production. Especially at high light, the HCO_3_^–^ influx can significantly exceed the rate of photosynthesis, with the ‘excess’ inorganic carbon lost as CO_2_ in (for example) the cyanobacterium *Synechococcus* and the eustigmatophycean eukaryotic alga *Nannochloropsis* (Tchernov *et al.*, 1997, 1998, 2003). There are also cases of CO_2_ influx exceeding the organic C production, implying net HCO_3_^–^ efflux.

However, the general case with MIMS measurements is that of CO_2_ decrease, or at least no increase, in the light. Here the MIMS method can be used to estimate CO_2_ efflux in the light from the CO_2_ efflux immediately after the cessation of illumination (Badger *et al.*, 1994; Salon *et al.*, 1996a,b; Eichner *et al.*, 2015; Table 3 and Supplementary Table S1, available at *JXB* online). Badger *et al.* (1994) found a leakage of not more than 0.1 of net photosynthesis in low inorganic carbon-grown cells of *Synechococcus*, while for low CO_2_ grown *Chlamydomonas* the corresponding leakage is 0.5 at low inorganic C and 0.1 at high inorganic C. Again using *Synechococcus*, Salon *et al*. (1996a,b) and Salon and Canvin (1997) were able to distinguish CO_2_ efflux from HCO_3_^–^ efflux immediately after darkening; the total inorganic C efflux in the presence of carbonic anhydrase was measured, as was the CO_2_ efflux under non-equilibrium conditions, and the difference is the HCO_3_^–^ efflux. The CO_2_ efflux was only 0.08 of the maximum CO_2_ influx, while the HCO_3_^–^ efflux was 0.45 of the maximum HCO_3_^–^ influx. The CO_2_ permeability coefficient determined from the measurements and expressed in terms of the plasmalemma area was 3.10^–8^ m s–^1^, while it was 1.6–2.5 m s^–1^ in terms of the carboxysome area (Tables 1, 14). The HCO_3_^–^ permeability coefficient expressed in terms of the plasmalemma area is at most 1.4–1.7.10^–9^ m s^–1^ (Table 4); the value is an upper limit because the inside-negative electrical potential component was not used in the calculation (Salon *et al.* 1996b; see also Ritchie *et al.* 1996).

In the case of *Trichodesmium* the leakage (CO_2_ efflux:gross inorganic carbon uptake) calculated using MIMS is 0.3–0.7 for two CO_2_ levels and with or without NO_3_^–^ (Eichner *et al.*, 2015), as compared with values of 0.5–0.9 in previous work on this organism (see Kranz *et al.*, 2009, 2010) (Table 3).

The other main method for estimating leakage of CO_2_ from aquatic organisms expressing a CCM is from natural abundance ^13^C/^12^C of particulate organic matter gained by photolithotrophic growth and of the ^13^C/^12^C of external inorganic carbon species (Sharkey and Berry, 1985; Eichner *et al.*, 2015; Table 3 and Supplementary Table S1). This method is also used for estimating leakage of CO_2_ from terrestrial C_4_ plants (see above). Eichner *et al.* (2015) found a difference between the MIMS and the natural abundance ^13^C/^12^C estimates of leakage, with the latter method giving values of the 0.82 and 1.14. They point out that the values > 1 are theoretically impossible; Eichner *et al.* (2015) suggest kinetic fractionation between CO_2_ and HCO_3_^–^ in the cytosol and/or enzymatic fraction by the ‘energized, unidirectional carbonic anhydrase’ NDH-1_4_ as possible causes of the very high leakage estimates. An analogous role might be played by the LCIA/LCIB system in *C. reinhardtii* (Wang and Spalding, 2014), so that estimates of leakage from carbon isotope ratios may be too high in *Chlamydomonas* and possibly in other eukaryotic algae as well. This possibility is acknowledged in Table 3 and Supplementary Table S1 (available at *JXB* online).

The mean value for the leakage determined by MIMS for cyanobacteria and eukaryotic algae in Supplementary Table S1 is, as indicated in Table 3, respectively 0.407±0.214 (standard deviation, *n*=5) and 0.36±0.16 (standard deviation, *n*=14). The mean values for hornworts with CCMs and C_4_ terrestrial flowering plants are 0.263±066 (standard deviation, *n*=3) and 0.260±0.106 (standard deviation, *n*=20), respectively. There is a trend (not significant) for lower fractional leakage in terrestrial C_4_ plants and hornworts than for cyanobacteria and eukaryotic algae.

As for C_4_ plants, so with cyanobacterial and algal CCMs: the prediction is an increasing fraction of the inorganic C pumped into the intracellular pool being lost as CO_2_ efflux with decreasing incident photosynthetically active radiation, and that algae relying on diffusive CO_2_ entry from the medium to Rubisco would be more common in low-irradiance habitats (review by Raven *et al.* 2000). The limited data available agree with these predictions (Raven *et al.*, 2000, 2002; Burkhardt *et al.*, 2001; de Araujo *et al.*, 2011; Cornwall *et al.*, 2015; see Table 3). Turning to temperature, Raven and Beardall (2014) show that algal CCMs occur at lower temperatures than does terrestrial C_4_ photosynthesis. Kranz *et al.* (2015) showed that the energy cost of algal CCMs decreased at low temperatures; it is not known if this is the case for terrestrial C_4_ photosynthesis.

## Leakage from the photorespiratory carbon oxidation cycle(s)

Tcherkez (2013) gives an excellent critique of the CO_2_ fluxes associated with C_3_ photosynthesis, photorespiration, and respiration. With a carboxylase:oxygenase ratio of Rubisco *in vivo* in a C_3_ plant in the present atmosphere of 3:1, CO_2_ production in the photorespiratory carbon oxidation cycle is 0.167 of gross CO_2_ assimilation in photosynthesis (Raven, 1972a,b; Tcherkez, 2013). There is about 15% recycling of the photorespiratory CO_2_ and ‘dark’ respiratory CO_2_ production in photosynthesizing structures (Raven, 1972a,b; Tcherkez, 2013), so the CO_2_ release into the environment as a fraction of gross photosynthesis is 0.167×0.85 or 0.14; it is likely that an upper limit is 0.20. This is at the low end of the range for leakage in C_4_ plants and in algae CCMs (Table 3).

The various C_3_–C_4_ intermediate flowering plants have photosynthetic gas exchanges that show varying mixtures of C_3_ and C_4_ characteristics (Hylton *et al.*, 1988; Rawsthorne *et al.*, 1988a,b; von Caemmerer, 1989; Rawsthorne and Hylton, 1991; Morgan *et al.*, 1993). This work shows the expression of most or all the glycine decarboxylase activity, and some of the Rubisco carboxylase–oxygenase, in bundle sheath cells. This location of the decarboxylase of the photorespiratory carbon oxidation cycle, with some Rubisco, in tightly packed bundle sheath cells increases recycling of CO_2_ from glycine decarboxylase by the carboxylase activity of Rubisco relative to leakage of CO_2_ back to the intercellular spaces.

CCMs increase the steady-state CO_2_:O_2_ ratios at the site of Rubisco activity; this decreases the ratio of Rubisco oxygenase activity to that of Rubisco carboxylase activity. The decreased rate of production of phosphoglycolate involves a decreased rate of the pathway(s) converting phosphoglycolate into phosphoglycerate and triose phosphate that can be used in core metabolism and/or complete oxidation to CO_2_ (Eisenhut *et al.*, 2008; Hagemann *et al.*, 2010; Young *et al.*, 2011; Raven *et al.*, 2012). Even this low flux is essential, since deletion of all three of the pathways of phosphoglycolate metabolism (photorespiratory carbon oxidation cycle; tartronic semialdehyde pathway; complete oxidation via oxalate) is lethal (Eisenhut *et al.*, 2008; Hagemann *et al.*, 2010; Raven *et al.*, 2012). Comparable work has not been yet been carried out in photosynthetic eukaryotes with CCMs where, at least in embryophytes, the pathway of phosphoglycolate metabolism is the photorespiratory carbon oxidation cycle. However, it is known that the C_4_ and CAM CCMs decrease the rate of phosphoglycolate synthesis and flux through the photorespiratory carbon oxidation cycle relative to what occurs in otherwise comparable C_3_ plants.

## Conclusions

Quantifying the flux of CO_2_ into and out of cells is difficult. All known irreversible decarboxylases produce CO_2_; CO_2_ is also the product/substrate of enzymes that can act as carboxylases and decarboxylases. Whether reversible of irreversible, decarboxylases produce CO_2_, which can potentially leak out of cells. Some irreversible carboxylases also have CO_2_ as their substrate; others use HCO_3_^–^.

There is still controversy as to the relative role of permeation through the lipid bilayer and of movement through membrane proteins such as CO_2_-selective aquaporins in the downhill, non-energized, movement of CO_2_. Such movement is involved in CO_2_ entry in terrestrial and aquatic organisms with C_3_ physiology and biochemistry, as well as terrestrial C_4_ plants and all CAM plants. Although there is also some evidence of active CO_2_ transport at the plasmalemma of algae, downhill CO_2_ transport is part of some mechanisms involved in the use of external HCO_3_^–^ and CCM function. Further work is needed to test the validity of the mechanism based on localized surface acidification in marine macrophytes, and on HCO_3_^–^ conversion to CO_2_ in the thylakoid lumen.

HCO_3_^–^ active influx at the plasmalemma underlies all cyanobacterial and some algal CCMs. HCO_3_^–^ can also enter chloroplasts of some algae, possible as part of a CCM. Leakage from the intracellular CO_2_ and HCO_3_^–^ pool of CCMs sometimes occurs as HCO_3_^–^, but typically occurs as CO_2_. Leakage from cyanobacterial and microalgal CCMs, and terrestrial C_4_ plants and hornworts with CCMs, usually involve half or less of the gross inorganic C entering in the CCM, but can be as high as 80%. CO_2_ leakage to the environment from photorespiration in C_3_ plants is less than 20% of gross photosynthesis. Leakage from terrestrial CAM plants, algal macrophytes, and vascular aquatic macrophytes with CCMs (C_4_, CAM, biophysical CCMs) has been less extensively examined. From what is known, CO_2_ leakage can be appreciable in many photoautotrophs with CCMs and increases the energetic cost of net inorganic carbon fixation (see Raven *et al.*, 2014).

## Supplementary data

Supplementary data are available at *JXB* online.

Table S1. Leakage of inorganic C from CCMs as a fraction of the inorganic C pumped into the intracellular pool.

Supplementary Data
