# Supplementary material for: The ins and outs of CO2
Source: J Exp Bot. 2015 Oct 14;67(1):1–13. doi: 10.1093/jxb/erv451 (PMC4682431; doi:10.1093/jxb/erv451)
Supplement: Supplementary Data [file supp_67_1_1__index.html]

The ins and outs of CO2 — The ins and outs of CO2 — The ins and outs of CO2 — Supplementary Data 

# The ins and outs of CO2

## Supplementary Data

Data files

- Supplementary Data - Supplementary Data
