# Supplementary material for: The ins and outs of CO2
Source: J Exp Bot. 2015 Oct 14;67(1):1–13. doi: 10.1093/jxb/erv451 (PMC4682431; doi:10.1093/jxb/erv451)
Supplement: Supplementary Data [file supp_erv451_supplementary_data.pdf]

SUPPLEMENTARY DATA

Supplementary Table S1.

Leakage of inorganic C from CCMs as a fraction of the inorganic C pumped into the intracellular pool for terrestrial C_4 flowering plants, hornworts, algae and cyanobacteria using a variety of techniques. Cyanobacteria and algae listed are all microscopic unicells, filaments or colonies. For C_3 flowering plants, leakage of CO_2 from photorespiration is less than 0.2 of gross CO_2 fixation (see main text).

Method	Organism	Leakiness	Reference
Radiolabelling-inorganic C pool	C_4 terrestrial flowering plants	0.15	Kromdijk *et al.* (2014)
Radiolabelling – leak rate	"	0.08-0.29	"
Quantum yield	"	-0.03-0.70	"
$\Delta^{13}C$ – dry matter	"	0.30-0.50	"
$\Delta^{13}C$ – online (high Irradiance values)	"	0.13-0.34	"
$\Delta^{13}C$ – dry matter	Three species of hornwort with CCMs	0.170, 0.304, -0.316	Meyer *et al.* (2008)
$\Delta^{13}C$ – dry matter	*Chlamydomonas reinhardtii* (Chlorophyceae)	0.42<br><br>0.05-0.18	Sharkey & Berry (1985);<br>Meyer *et al.* (2008, 2012)
MIMS: CO_2 efflux immediately upon darkening	*Synechococcus* (Cyanobacteria)	≤0.1	Badger *et al.* (1994)
"	*Chlamydomonas reinhardtii* (Chlorophyceae)	0.5 (low inorganic C)<br>0.1 (high inorganic C)	Badger *et al.* (1994)
"	*Synechococcus* (Cyanobacteria)	0.5	Salon *et al.* (1996)
"	*Phaeodactylum tricornutum*[1] (Bacillariophyceae)	0.09 (CO_2 0.1 x PAL)<br>0.36 (CO_2 10 x PAL)	Burkhardt *et al.* (2001)
"	*Thalassiosira weissflogii* (Bacillariophyceae)	0.01 (CO_2 0.1 x PAL)<br>0.28 (CO_2 10 x PAL)	Burkhardt *et al.* (2001)
"	*Emilania huxleyi* (Prymnesiophyceae)	0.60 (CO_2 0.1 x PAL)<br>0.38 (CO_2 5 x PAL)	Rost *et al.* (2006a)
"	*Phaeocystis globosa* (Prymnesiophyceae)	0.45 (CO_2 0.1 x PAL<br>0.23 (CO_2 5 x PAL)	Rost *et al.* (2006a)
"	*Skeletonema costatum* (Bacillariophyceae)	0.37 (CO_2 0.1 x PAL)<br>0.15 (CO_2 5 x PAL)	Rost *et al.* (2006a)
"	*Ceratium linearum*[2] (Dinophyceae)	0.6-0.8 (slightly higher at low [CO_2])	Rost *et al.* (2006b)
"	*Heterocapsa triquetra*[3] (Dinophyceae)	0.1-0.2 (little effect of [CO_2])	Rost *et al.* (2006b)
"	*Prorocentrum minimum*[3] (Dinophyceae)	0.3-0.4 (little effect of [CO_2])	Rost *et al.* (2006b)
"	*Nitzschia navis-varingica* (Bacillariophyceae)<br>*Pseudo-nitzschia multiseries* (Bacillariophyceae)<br>*Stellarima stellaris* (Bacillariophyceae)	0.05 – 0.57 (little effect of growth pH; highest values at low [CO_2])<br>0.15-0.40 (little effect of growth pH; highest values at low [CO_2])<br>0.10-0.57 (little effect of growth pH; highest values at low [CO_2])	Trimborn *et al.* (2008)
"	*Trichodesmium* (Cyanobacteria)	0.24-0.41	Kranz *et al.* (2010)
$\Delta^{13}C$ – dry matter	*Trichodesmium* (Cyanobacteria)	0.57-0.92	Kranz *et al.* (2010)
MIMS; CO_2 efflux immediately upon darkening	*Leptolyngbya* (Cyanobacteria)	0.78 (low irradiance)<br>0.45 (high irradiance)	De Araujo *et al.* (2011)
"	*Synechocystis* sp. (Cyanobacteria)	0.09	Jiang *et al.* (2008)
"	*Alexandrium tamarense* (Dinophyceae)	0.44 (low CO_2) -0.63 (high CO_2)	Eberlein *et al.* (2014)
"	*Scrippsiella trochoidea* (Dinophyceae)	0.56 (low CO_2) -0.48 (high CO_2)	Eberlein *et al.* (2014)
"	*Trichodesmium* (Cyanobacteria)	0.30-0.61 (low CO_2) – 0.37-0.69 (high CO_2)	Eichner *et al.* (2015)
$\Delta^{13}C$ –dry matter	*Trichodesmium*[4] (Cyanobacteria)	0.82-1.08 (low CO_2) – 0.90-1.14 (high CO_2)	Eichner *et al.* (2015)

Table S1 Footnotes

[1]PAL = Present Atmospheric Level

[2]Grown at pH 8.5. MIMS values similar to those derived from $\Delta^{13}C$ – dry matter

[3]Grown at pH 9.1. MIMS values similar to those derived from $\Delta^{13}C$ – dry matter

[4]Values of leakage in excess of 1.0 are theoretically impossible. Reasons for these very high values are discussed by Eichner *et al.* (2015).

References

Badger MR, Palmqvist K, Yu JW. 1994. Measurements of CO_2 and HCO_3^- fluxes in cyanobacteria and microalgae during steady-state photosynthesis. Physiologia Plantarum **90,** 529-536.

Burkhardt S, Amoroso G, Riebesell U, Sültemeyer D. 2001. CO_2 and HCO_3^- uptake in marine diatoms acclimated to different CO_2 concentrations. Limnology and Oceanography **46,** 1378-1391.

de Araujo ED, Patel J, de Araujo C, Rogers SP, Short SM, Cambell DA, Espie GS. 2011. Physiological characterization and light response of the CO_2-concentrating mechanism in the filamentous cyanobacterium *Leptolyngbya* sp. CPPP 696. Photosynthesis Research **109,** 95-103.

Eberlein T, Van der Waal DB, Rost B. 2014. Differential effects of ocean acidification on carbon acquisition in two bloom-forming dinoflagellate species. Physiologia Plantarum **151,** 468-479.

Eichner M, Thoms S, Kranz SA, Rost B. 2015. Cellular inorganic carbon fluxes in *Trichodesmium*: a combined approach using measurements and modelling. Journal of Experimental Botany **66,** 749-759.

Jiang H-B, Cheng H-H, Gao K-S, Qiu B-S. 2013. Inactivation of Ca^{2+}/H^+ exchanger in *Synechocystis* sp. strain PCC 6803 promotes cyanobacterial calcification by upregulation of CO_2-concentrating mechanisms. Applied and Environmental Microbiology **79,** 4048-4055.

Kranz SA, Levitan O, Richter K-U, Prasil O, Berman-Frank I, Rost B. 2010. Combined effects of CO_2 and light on the N_2 fixing cyanobacterium *Trichodesmium* IMS101: physiological responses. Plant Physiology **154,** 334-345.

Kromdijk J, Ubierna N, Cousins AB, Griffiths H. 2014. Bundle-sheath leakiness in C_4 photosynthesis: a careful balancing act between CO_2 concentration and assimilation. Journal of Experimental Botany **65,** 3443-3457.

Meyer M, Seibt U, Griffiths H. 2008. To concentrate or ventilate. Carbon acquisition, isotope discrimination and physiological ecology of early land plant life forms. Philosophical Transactions of the Royal Society of London B **363,** 2767-2778.

Meyer M, Genkov T, Skepper JN, Jouhet J, Mitchell MC, Spreitzer RJ, Griffiths H. 2012. Rusbisco small-subunit α-helices control pyrenoid formation in *Chlamydomonas.* Proceedings of the National Academy of Sciences USA **109,** 19474-19479.

Rost B, Riebesell U, Sültemeyer D. 2006a. Carbon acquisition of marine phytoplankton. Effect of photoperiod length. Limnology and Oceanography **51,** 12-20.

Rost B, Richter K-U, Riebesell U, Hansen PJ. 2006b. Inorganic carbon acquisition by red tide dinoflagellates. Plant Cell and Environment **29,** 810-822.

Salon C, Mir NA, Canvin DT. 1996. Influx and efflux of inorganic carbon in *Synechococcus* UTEX 625. Plant Cell and Environment **19,** 245-259.

Sharkey TD, Berry JA. 1985. Carbon isotope fractionation of algae influenced by an inducible CO_2-concentrating mechanism. In: Lucas WJ, Berry JA, eds. *Inorganic carbon uptake by aquatic photosynthetic organisms.* Rockville: American Society of Plant Physiologists, 389-401.

Trimborn S, Lundhom N, Thoms S, Richter K-U, Kock B, Hansen PJ, Rost B. 2008. Inorganic carbon acquisition in potentially toxic and non-toxic diatoms: the effect of pH-induced changes in seawater carbonate chemistry. Physiologia Plantarum **133,** 92-105.
